# Supplementary material for: Strong Root System Enhances Waterlogging Resilience in Barley (Hordeum vulgare) at the Early Stage Stress
Source: Plants (Basel). 2026 Jan 2;15(1):134. doi: 10.3390/plants15010134 (PMC12787377; doi:10.3390/plants15010134)
Supplement: Supplementary file 1 [file plants-15-00134-s001.zip › plants-4020720-supplementary.pdf]

Supplementary Table S1. The list of barley varieties used in this study.

No.	Genotype	Origin	No.	Genotype	Origin
FPQ-1	Yangzhou 27	China(Jiangsu)	FPQ-18	Xiumai 3 hao	China(Zhejiang)
FPQ-2	Maodamai	China(Shanxi)	FPQ-19	Jialing 6 hao	China(Zhejiang)
FPQ-3	Zidamai	China(Jiangsu)	FPQ-20	Jia-8	China(Zhejiang)
FPQ-4	Ziganliuleng	China(Jiangsu)	FPQ-21	Suyinmai 3 hao	China(Jiangsu)
FPQ-5	Yuhangsilengmaodamai	China(Zhejiang)	FPQ-22	Edamai 072	China(Hubei)
FPQ-6	Zhunai	China(Xizang)	FPQ-23	Zhenongda 7 hao	China(Zhejiang)
FPQ-7	Goumangqingke	China(Xizang)	FPQ-24	Hua 30	China(Shanghai)
FPQ-8	Qilianhongjiaoni	China(Qinghai)	FPQ-25	Yang QS	China(Jiangsu)
FPQ-9	Songjianglaotuoxu	China(Shanghai)	FPQ-26	Yuyaoxiangtian 2 leng	China(Zhejiang)
FPQ-10	Shangyuzisileng	China(Zhejiang)	FPQ-27	Fergus	Canada
FPQ-11	Yueqingliulengzidamai	China(Zhejiang)	FPQ-28	Hua 9	China(Hubei)
FPQ-12	Guangtoumimai	China(Hubei)	FPQ-29	Yangsimai 5 hao	China(Jiangsu)
FPQ-13	Guoluodongqingke	China(Xizang)	FPQ-30	Yangyin 2 hao	China(Jiangsu)
FPQ-14	Emai 32380-9	China(Hubei)	FPQ-31	Sunong 16	China(Jiangsu)
FPQ-15	Yangnongpi 5 hao	China(Jiangsu)	FPQ-32	Naso Nijo	Japan
FPQ-16	Zheyuan 18 hao	China(Zhejiang)	FPQ-33	TX9425	China(Jiangsu)
FPQ-17	Zhepi 8 hao	China(Zhejiang)			

Supplementary Table S2. The list of primer sequences used in qRT-PCR.

Gene name	Primers sequence
HvTCP20-F	ACTGTATGCACGCAGCTATG
HvTCP20-R	GAACCGGAGGGAATTCTGGC
HvPLT2-F	TGGGAAGAAACGGCGCTTTA
HvPLT2-R	GATGGACTTCCTCGCGACAG
HvPILS2-F	GCCACCTTCAAGCTGCTCA
HvPILS2-R	ATGACGGTGAAGCGGAAGAA
HvSCR-F	CCATGTCACGGGGGATCTCT
HvSCR-R	TTAACCTCCGTGGCGATCTG
HvSHR-F	CGAGGGCTTACGGTTCTTCT
HvSHR-R	TGAATGGCACCGGTGAGAAC
HvUBI-F	AAGCAGCCAGAATGTACAGCGAGAAC
HvUBI-R	GGTACAGACCAGCAAAGCCAGAAATG
